# Cytoprotective effect of palm kernel cake phenolics against aflatoxin B1-induced cell damage and its underlying mechanism of action

**DOI:** 10.1186/s12906-015-0921-z

**Published:** 2015-10-30

**Authors:** Ehsan Oskoueian, Norhani Abdullah, Idrus Zulkifli, Mahdi Ebrahimi, Ehsan Karimi, Yong Meng Goh, Armin Oskoueian, Majid Shakeri

**Affiliations:** Institute of Tropical Agriculture, Univeristi Putra Malaysia, 43400 Serdang, Selangor Malaysia; Agricultural Biotechnology Research Institute of Iran (ABRII), East and North-East Branch, P.O.B. 91735/844, Mashhad, Iran; Department of Biochemistry, Faculty of Biotechnology and Biomolecular Sciences, University Putra Malaysia, 43400 Serdang, Selangor Malaysia; Department of Veterinary Preclinical Sciences, Faculty of Veterinary Medicine, Universiti Putra Malaysia, 43400 Serdang, Selangor Malaysia; Department of Biochemistry and Biophysics, Mashhad Branch, Islamic Azad University, Mashhad, Iran; Department of Crop Science, Faculty of Agriculture, Universiti Putra Malaysia, 43400 Serdang, Selangor Malaysia; Ferdowsi University of Mashhad, International Branch, Mashhad, Iran

**Keywords:** Aflatoxin B1, Cytoprotection, Oxidative stress, Antioxidant enzyme, Molecular mechanism, Apoptosis

## Abstract

**Background:**

Palm kernel cake (PKC), a by-product of the palm oil industry is abundantly available in many tropical and subtropical countries. The product is known to contain high levels of phenolic compounds that may impede the deleterious effects of fungal mycotoxins. This study focused on the evaluation of PKC phenolics as a potential cytoprotective agent towards aflatoxin B1 (AFB1)-induced cell damage.

**Methods:**

The phenolic compounds of PKC were obtained by solvent extraction and the product rich in phenolic compounds was labeled as phenolic-enriched fraction (PEF). This fraction was evaluated for its phenolic compounds composition. The antioxidant activity of PEF was determined by using 1,1-diphenyl-2-picryl-hydrazil scavenging activity, ferric reducing antioxidant power, inhibition of ß-carotene bleaching, and thiobarbituric acid reactive substances assays. The cytotoxicity assay and molecular biomarkers analyses were performed to evaluate the cytoprotective effects of PEF towards aflatoxin B1 (AFB1)-induced cell damage.

**Results:**

The results showed that PEF contained gallic acid, pyrogallol, vanillic acid, caffeic acid, syringic acid, epicatechin, catechin and ferulic acid. The PEF exhibited free radical scavenging activity, ferric reducing antioxidant power, ß-carotene bleaching inhibition and thiobarbituric acid reactive substances inhibition. The PEF demonstrated cytoprotective effects in AFB1-treated chicken hepatocytes by reducing the cellular lipid peroxidation and enhancing antioxidant enzymes production. The viability of AFB1-treated hepatocytes was improved by PEF through up-regulation of oxidative stress tolerance genes and down-regulation of pro-inflammatory and apoptosis associated genes.

**Conclusions:**

The present findings supported the proposition that the phenolic compounds present in PKC could be a potential cytoprotective agent towards AFB1 cytotoxicity.

## Background

Aflatoxins produced by *Aspergillus* species, can be ubiquitously found in many foodstuffs. The aflatoxin B_1_ (AFB1) produced by both *Aspergillus flavus* and *Aspergillus parasiticus* is considered the most toxic among the mycotoxins [1]. Upon ingestion, this mycotoxin causes hepatotoxicity and alters the blood and immunological parameters [2]. The AFB1 triggers the generation of reactive oxygen species (ROS) in different organs, impairs the antioxidant/pro-oxidant imbalance, elevates lipid peroxidation and damages biological molecules including lipids, proteins and DNA. The combination of these manifestations leads to oxidative stress and initiates the malfunction of the liver, which is the main detoxifying organ in the body [3, 4].

Recent studies suggested that plant phenolics and flavonoids are capable of adsorbing mycotoxins and alleviating their side effects in animals. The adsorption of toxic metabolites including aflatoxins, ochratoxins and fumonisins, boosts liver function and consequently enhances animal health and production [3, 5–7]. In this respect, phenolics, including sylimarin [8], rosmarinic acid [9], carnosic acid [10], catechins [11, 12] hesperidin [13], thymol [3] and quercetin [6] have been found to posses cytoprotective effects. However, none of these compounds have been commercialised as AFB1 cytoprotective agents due to their limited supply. Consequently, easily available sources of phenolic compounds such as agro-industrial by-products should be considered as an alternative source of these metabolites. The palm kernel cake (PKC), the residue from the kernel during oil extraction, would offer a sustainable source of phenolic compounds as the by-product is abundantly produced in countries like Indonesia, Malaysia, Philippines, Thailand, India, Nigeria, Colombia and Ivory Coast [14, 15].

Some reports are available on the phenolic compounds present in the palm oil and leaf [16], but information concerning the characteristics and function of phenolic compounds present in the PKC is rather limited and inconclusive. Therefore, we hypotised that the phenolics present in PKC may have antioxidant potential to impede the AFB1 cytotoxixity effects. In this regard, chicken hepatocytes, as one of the most sensitive cells to AFB1 manifestations, were used to evaluate the cytoprotective effects of PKC phenolics and to determine the underlying mechanisms of protection against AFB1 cytotoxicity.

## Methods

### Agriculture by-product

The expeller type PKC was obtained from Oil Mill Sdn Bhd., Dengkil, Selangor, Malaysia. The samples were freeze dried (Labconco, Kansas City, USA) and ground (mesh 100) using a laboratory grinder and stored at –20 °C before used.

### Extraction of phenolic compounds

The reflux extraction technique was applied to extract the phenolic compounds in PKC following the method as described by Crozier et al*.* [17]. Briefly, 5 g of dried PKC powder were transferred into a 500 ml round-bottom flask and 160 ml of methanol were added, followed by 40 ml of 6 M HCL solution. The flask was then heated for two hours at 90 °C and the mixture was filtered (No. 1, Whatman, England). The methanol was removed under vacuum using a Rotary Evaporator (Rotavapour Buchii, Flawil, Switzerland) and the aqueous phase was then washed using n-hexane (20 ml) and subjected to liquid-liquid extraction with diethyl ether (3 × 20 ml) and ethyl acetate (3 × 20 ml). The organic solvents were then removed by using a Rotary Evaporator (Buchii, Switzerland) at 50 °C. The dried fraction obtained was weighed and reconstituted in dimethyl sulfoxide and labeled as PKC phenolic-enriched fraction (PEF).

### Total phenolic contents

The total phenolic content (TPC) of PEF was determined according to the method described by Ismail et al*.* [18]. The PEF solution (0.5 ml) was mixed with 2.5 ml Folin-Ciocalteu reagent (previously diluted with water 1:10, v/v) and 2 ml sodium carbonate solution (7.5 %, w/v) and subsequently incubated for 90 min in the dark. The absorbance of the mixture was determined using a spectrophotometer (Molecular Devices, Sunnyvale, CA, USA) at 765 nm and the result was expressed as milligram of gallic acid equivalent (GAE) per gram of dried PEF.

### Analyses of phenolic compounds by HPLC

To determine the quantity and types of phenolic compounds, the PEF was analysed by a high performance liquid chromatograph (Waters, Milford, MA, USA) equipped with an analytical column (Intersil ODS-3, 5 μm 4.6x150 mm, Gl Science Inc) as described by Karimi et al. [19].

The mobile phase consisted of deionized water (solvent A) and acetonitrile (solvent B). The pH of deionized water was adjusted to 2.5 with trifluoroacetic acid. The column was equilibrated by 85 % solvent A and 15 % solvent B. The elution was established by increasing the ratio of solvent B from 15 % to 85 % in 50 min. Then, the solvent B was decreased to 15 % in the next 5 min and this ratio was maintained for an extra 10 min for re-equilibration. The flow rate was 0.6 ml/min and the phenolic compounds were detected at 280 nm. For quantification of phenolic compounds, a calibration curve was prepared by injection of different standard compounds. The results were expressed as milligram of each phenolic compound per gram of dried PEF.

### Antioxidant activity

#### Radicals scavenging activity

The 1,1-diphenyl-2-picryl-hydrazil (DPPH) free radicals were used to evaluate the radical scavenging activity of PEF following the method described by Gulcin et al*.* [20]. The PEF solution was diluted in methanol and 1 ml of the solution was mixed with DPPH methanolic solution (0.1 mM). The mixture was incubated in a dark condition, at room temperature for 30 min. The absorbance of the mixture was read using a spectrophotometer (Molecular Devices, Sunnyvale, CA, USA) at 517 nm. The gallic acid was used as the standard antioxidant and the free radical scavenging activity of the PEF was calculated as follows:

$$ \mathsf{Radical}\kern0.5em \mathsf{scavenging}\ \mathsf{activity}\left(\%\right)=\left[\left(\mathsf{a}\hbox{-} \mathsf{b}\right)/\left(\mathsf{a}\right)\right]\times 100 $$

Radical scavenging activity (%) = [(a-b) / (a)] × 100 a= Absorbance of negative control; b= Absorbance of sample

### Ferric reducing antioxidant power (FRAP)

The ferric reducing antioxidant power of PEF fraction was evaluated according to the method described by Yen and Chen [21]. Briefly, 1 ml of PEF solution, 2.5 ml of potassium phosphate buffer (0.2 M, pH 6.6) and 2.5 ml of potassium ferricyanide (1 %, w/v) were added to the test tube, and the mixture was incubated at 50 °C for 20 min. In order to stop the reaction, 2.5 ml trichloroacetic acid (10 %, w/v) were added and the mixture was centrifuged at 3000 × g for 15 min. The upper layer of solution (2.5 ml) was transferred to the test tube containing 2.5 ml of distilled water and 0.5 ml FeCl_3_ (0.1 %, w/v). The solutions were mixed properly, and the absorbance of the reaction was determined at 700 nm (Molecular Devices, Sunnyvale, CA, USA) . Gallic acid was used as the reference antioxidant and the ferric reducing antioxidant power of samples was calculated using the following formula.

$$ \mathsf{Antioxidant}\ \mathsf{activity}\ \left(\%\right) = \left[\left(\mathsf{a}\hbox{-} \mathsf{b}\right)\ /\ \left(\mathsf{a}\right)\right] \times 100 $$

Antioxidant activity (%) = [(a-b) / (a)] ×100a= Absorbance of negative control; b= Absorbance of sample

### Inhibition of ß-carotene bleaching

The ß-carotene bleaching assay was used to determine the antioxidant activity of PEF according to the method described by Ismail et al*.* [18]. Three milliliter of ß-carotene solution (5 mg ß-carotene/50 ml chloroform), linoleic acid (40 mg) and Tween 20 (400 mg) were mixed thoroughly and then a stream of nitrogen gas was passed to dry the mixture. The ß-carotene-linoleic acid emulsion was prepared by adding 100 ml of ultra-pure water. Then, to 1.5 ml of ß-carotene-linoleic acid emulsion, 20 μl of PEF solution were added and the mixture was incubated in a water bath (50 °C, 60 min). At the end of the incubation, the reacting mixtures were cooled and, the absorbance was read at 470 nm using a spectrophotometer (Molecular Devices, Sunnyvale, CA, USA). The gallic acid was used as the standard in this assay. The following formula was applied to calculate the antioxidant activity of PEF.

$$ \mathsf{Antioxidant}\ \mathsf{activity}\ \left(\%\right) = \left[\left(\mathsf{R}\mathsf{D}\mathsf{c}\ \hbox{--}\ \mathsf{R}\mathsf{D}\mathsf{s}\right)\ /\left(\mathsf{R}\mathsf{D}\mathsf{c}\right)\right] \times \mathsf{100} $$

Antioxidant activity (%) = [(RDc – RDs) /(RDc)] ×100

RDc= Rate of degradation in the control: [(a/b) /60]; RDs= Rate of degradation in the sample: [(a/b) /60]; a = Initial absorbance of the sample; b = Absorbance after 60 min of incubation

### Thiobarbituric acid reactive substances assay (TBARS)

The TBARS assay was used to evaluate the potential of PEF in preventing the oxidation of linoleic acid under oxidative condition, according to the method described by Hendra et al*.* [22]. The PEF (4 mg) was dissolved in 4 ml of absolute ethanol and then 4.1 ml of 2.5 % linoleic acid in 99 % ethanol, 8 ml of phosphate buffer (0.05 M, pH 7) and 3.9 ml of ultra-pure water were added. The mixture was transferred to the 15 ml screw cap test tube, capped tightly and incubated in a 40 °C oven for 6 days. At the end of the incubation period, 2 ml of sample solutions, aqueous solution of trichloroacetic acid [1 ml of 20 % (w/v)] and aqueous thiobarbituric acid [2 ml, 0.67 % (w/v)] were mixed in a screw cap test glass tube and incubated in boiling water bath for 10 min. The tube was cooled to room temperature and centrifuged at 3000 × g for 20 min. The absorbance of the supernatant was determined using a spectrophotometer (Molecular Devices, Sunnyvale, CA, USA) at 532 nm.

The antioxidant activity was reported as:

$$ \mathsf{Percent}\ \mathsf{inhibition} = \left[\left(\mathsf{a}\hbox{-} \mathsf{b}\right)\ /\ \left(\mathsf{a}\right)\right] \times \mathsf{100} $$

Percent inhibition = [(a-b) / (a)] × 100 a = Absorbance of the control reaction; b: Absorbance of the sample reaction

### Isolation and culture of primary chicken hepatocytes

The chicken hepatocytes were isolated using the 2-step collagenase method as described by Wang et al*.* [23]. Five-week-old male chickens were treated by intra-peritoneal injection of natrium thiopenthal (45 mg/kg) and heparin (1500 U/kg). The abdominal cavity was opened after full anaesthesia. The liver was perfused with different buffers as described by Wang et al*.* [23] and then the liver was excised and digested using 0.5 mg/ml of collagenase type IV for 25 min at 37 °C. The William’s E medium (Gibco, Grand Island, NY) containing 5 % chicken serum and 2 mg/ml bovine serum albumin (BSA) was used to stop the digestion. The cells were passed through 100, 60 and 30 μm sieves and subsequently incubated with red blood cell lysis buffer (Sigma–Aldrich, St. Louis, MO, USA) and rewashed using William’s E medium containing chicken serum to eliminate the red blood cells. The accuracy of the isolated hepatocytes were confirmed according to morphological characteristics. Cells were cultured in William’s E medium supplemented with 100 U/ml of penicillin-streptomycin, 10 μg/ml insulin and 5 % chicken serum. The cells were incubated at 37 °C with 5 % CO_2_ in a humidified incubator. The approval of Animal Use and Care Committee (ACUC),, Faulty of Medicine and Health Sciences, University Putra Malaysia for this procedure was obtained.

### Cytotoxicity assay

The cytotoxicity effect of PEF on chicken hepatocytes was determined using MTT assay [24]. The cells were grown in each well of 96-well plates with the density of 5 × 10^3^ cells/ 100 μl of the medium. The cells were pre-treated with the serial concentrations of PEF (0, 5, 10, 20, 40 μg/ml) and gallic acid (10 μM or 1.7 μg/ml) as positive control. The cells incubated in a medium devoid of PEF (0 μg/ml) was considered as a negative control. The cells were incubated for 24 h, and then the media were replaced with the fresh media containing 5 μM of AFB1 (Cayman Chemical Company, Ann Arbor, MI, USA) and incubated for another 48 h. Finally, the viability of the cells was determined by using 3–(4,5–Dimethylthiazol-2-yl)-2,5-Diphenyltetrazolium Bromide (MTT) assay. The experiment was conducted in triplicates.

### Antioxidant enzyme assay

The cells were treated as mentioned earlier in the cytotoxicity assay. Upon treatments, cells were rinsed with ice-cold phosphate-buffered saline (PBS, 0.1 M, pH 7.4) for three times. Then, 6 ml of PBS were added, cells were scraped and transferred into 15 ml centrifuge tube. The cells were centrifuged at 250 × g for 20 min at 4 °C and the supernatant was discarded. The cells were lysed immediately at 4 °C using 150 μl of lysis buffer (0.5 % Triton x-100, 2 mM ETDA in 20 mM Tris–HCl pH 7.5) and then sonicated for 10 s using a sonicator (Hielscher, Teltow, Germany). Then lysates were centrifuged at 2800 × g for 10 min at 4 °C. The supernatant was collected to determine the antioxidant enzymes activity. The activities of superoxide dismutase (SOD), catalase (CAT) and glutathione reductase (GR) were determined by using enzyme kits from Nanjing Jiancheng Bioengineering Institute (Nanjing, China) according to the instructions provided by the kits. The results were expressed as enzyme activity/g protein (U/ g protein) of the cells.

### Lipid peroxidation assay

The lipid peroxidation in the chicken hepatocytes was determined by measuring the malondialdehyde (MDA) using thiobarbituric acid reactive substances (TBARS) [25]. The treatments were similar to the cytotoxicity test. Treated cells were rinsed with phosphate-buffered saline (PBS, 0.1 M) for three times and scraped. The scraped cells were suspended in 4 ml of potassium chloride (1 %) and homogenised using an Ultra-Turrax homogeniser (Wilmington, NC, USA) at 20,000 rpm for 25 s while kept on ice. Then, 300 μl distilled water, 200 μl of homogenised cells, 35 μl of BHT, 165 μl sodium dodecyl sulphate (SDS) and 2 ml TBA were added into the screw cap test tube. The solution was mixed and heated at 90 °C for 60 min. The solution was cooled immediately and 3 ml of n-butanol were added, shaken for 30 s and centrifuged at 2800 × g for 10 min. The absorbance of n-butanol fraction was recorded at 532 nm by a spectrophotometer (Molecular Devices, Sunnyvale, CA). The 1,1,3,3-tetraethoxypropane was used to construct the standard curve.

### Gene expression analyses

The hepatocytes were cultured and treated as mentioned in the cytotoxicity assay. Treated cells were rinsed with phosphate-buffered saline (PBS, 0.1 M, pH 7.2) for two times and scraped. Total RNA was extracted from cells using a RNasey mini kit (Qiagen, Valencia, CA, USA). The total RNA was converted to cDNA through reverse transcript PCR technique using Maxime RT Permix kit (iNtRON Biotechnology, Sungnam, Korea). The expression of nuclear factor kappa-light-chain-enhancer of activated B cells (NF-kB), nitric oxide synthase (iNOS), tumor necrosis factor alpha (TNF-α), interleukin-1 beta (IL1ß), interleukin-6 (IL6), bax, bcl2, glyceraldehyde 3-phosphate dehydrogenase (GAPDH) and β-Actin genes (Table 1) were analysed by Real time PCR thermocycler (Bio-Rad, CA, USA) using iQ SYBR Green Supermix (Bio-Rad, CA, USA). The amplification conditions were optimised for all genes as follows: 95 °C for 5 min (1X), then 95 °C for 20 s, then 58 °C for 20 s and 72 °C for 25 s (35X). The expressions of genes were normalised to GAPDH and ß-actin as housekeeping genes according to Vandesompele et al*.* [26] method using CFX manager software version 2 (Bio-Rad Laboratories). All the real time PCR amplifications were conducted in triplicates.

**Table 1:** The primer characteristics used for the gene expression analysis

Genes		Sequences (5′ to 3′)	References
NF-kB	F	gaaggaatcgtaccgggaaca	[39]
	R	ctcagagggccttgtgacagtaa	
iNOS	F	gaacagccagctcatccgata	[40]
	R	cccaagctcaatgcacaactt	
TNF-α	F	tgtgtatgtgcagcaacccgtagt	[2]
	R	ggcattgcaatttggacagaagt	
IL1ß	F	tgggcatcaagggctaca	[41]
	R	tcgggttggttggtgatg	
IL6	F	caaggtgacggaggaggac	[41]
	R	tggcgaggagggatttct	
Bax	F	tcctcatcgccatgctcat	[42]
	R	ccttggtctggaagcagaaga	
Bcl2	F	gatgaccgagtacctgaacc	[42]
	R	caggagaaatcgaacaaaggc	
GAPDH	F	gtcagcaatgcatcgtgca	[40]
	R	ggcatggacagtggtcataaga	
β-Actin	F	acacggtattgtcaccaact	[41]
	R	taacaccatcaccagagtcc	

### Western blot analysis

The expression of 70 kilodalton heat shock protein (Hsp70), caspase-3 and GAPDH proteins were determined by Western blot analysis. The chicken hepatocytes were cultured and treated as described in the cytotoxicity assay. The cells were trypsinised and washed with ice-cold PBS (0.1 M, pH 7.2) and the cells were immediately lysed at 4 °C using 150 μl of lysis buffer (0.5 % Triton x-100, 2 mM ETDA in 20 mM Tris–HCl pH 7.5) containing 15 μl/ml of protease inhibitor (ProteoBlock Protease Inhibitor Cocktail, Fermentas, MD, USA). In order to facilitate the extraction of proteins, the cells were sonicated for 15 s using a sonicator (Hielscher, Teltow, Germany) and incubated on ice for 20 min. In order to collect the supernatant, cell lysates were centrifuged at 15,000 × g for 25 min and the protein concentration of the supernatant was determined using a Protein Assay kit (Bio-Rad, CA, USA). The protein (25 μg) was denatured at 95 °C for 5 min and subjected to electrophoresis using Tris-glycine polyacrylamide gel. The Hoefer Semi-Dry Transfer Unit was used to transfer the protein to a PVDF membrane, and the membrane was washed using Odyssey Blocking Buffer (LI-COR, Lincoln, NE, USA). The membrane was incubated in heat shock protein 70 (Hsp70) (Biorbyt orb10848), nuclear factor (erythroid-derived 2)-like 2 (Nrf2) (Biorbyt orb11165), caspase-3 (Biorbyt orb10237) and GAPDH (Thermo Scientific MA1-4711) primary antibodies with dilution rates ranging from 1:500 up to 1:1000 overnight at 4 °C. The PBST (phosphate buffer saline and Tween 20, 0.05 %) was used to wash the membrane for three times. The IRDye 680 Goat Anti-Mouse or IRDye 800 CW Goat Anti-Rabbit secondary antibodies were applied to detect the target proteins by using the Odyssey Infrared Imaging System (LI-COR, Lincoln, NE, USA). The intensity of the bands were analysed by the Odyssey software.

### Apoptosis analysis by flow cytometry

The chicken hepatocytes were cultured at the density of 1 × 10^6^ cells per 75 cm^2^ flask and treated as mentioned in the cytotoxicity test. The cells were trypsinised and washed with ice-cold PBS (0.1 M, pH 7.2). Then, cells were stained using FITC Annexin V Apoptosis Detection Kit I (BD Biosciences Pharmingen, San Diego, CA, USA) according to the manufacturer’s instruction. The cell apoptosis was evaluated by flow cytometry (FACS-Canto II BD Biosciences) and the data were analysed using Diva software (BD Biosciences, Franklin Lakes, NJ, USA). The characteristics of viable cells were FITC Annexin V and PI negative, whereas early apoptotic cells to be FITC Annexin V positive and PI negative. The cells in late apoptosis or already dead were both FITC Annexin V and PI positive.

### Statistical analysis

All the data obtained from this study were analysed in a completely randomised design using the GLM procedure of SAS [27]. The differences between means were determined by Duncan’s Multiple Range Test and considered significant at *p* < 0.05. All measurements were performed in triplicate samples and carried out independently at least three times.

## Results

### Extraction yield and total phenolic contents

The yield of PEF was 9.0 ± 0.86 g/100 g dry PKC, and the amount of total phenolics was 658.3 ± 26.32 mg gallic acid equivalents (GAE) /g dried PEF (Table 2).

**Table 2:** The extraction yield and total phenolic content of phenolic-enriched fraction (PEF)

Items	PEF
Extraction yield (g/100 g DM ^a^)	9.0 ± 0.86
Total phenolic content ^b^ (mg/g DM)	658.3 ± 26.32

All data are presented as means (± S.E.M) of at least three replicates (*n* = 3)

*S.E.M* Standard error of the means

^a^DM: Dry matter

^b^mg gallic acid equivalents (GAE)/g dry matter PEF

### Analyses of phenolic compounds by HPLC

**Fig. 1:**
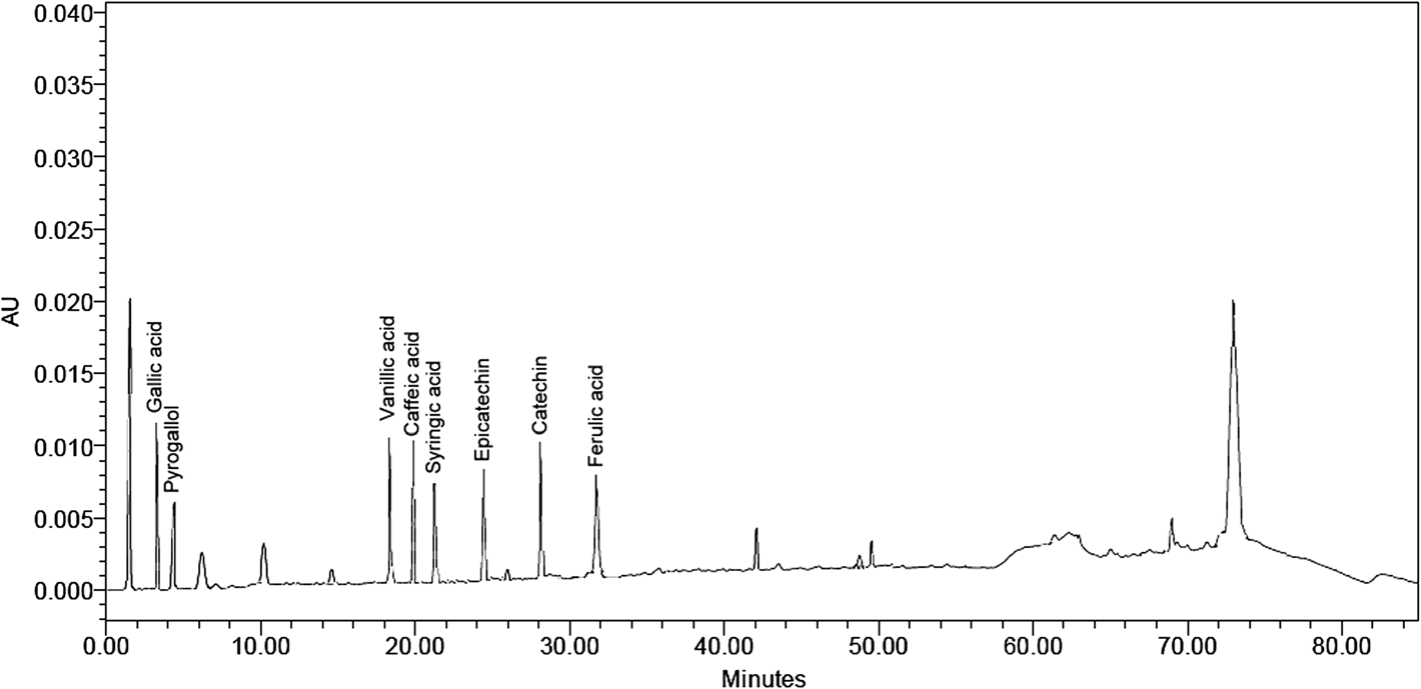
The HPLC chromatogram of phenolic acids present in phenolic-enriched fraction (PEF) obtained from PKC detected at 280 nm

As shown in Fig. 1, the PEF contained phenolic acids, including gallic acid, pyrogallol, vanillic acid, caffeic acid, syringic acid, epicatechin, catechin and ferulic acid with the concentrations ranging from 6.9 to 13.2 mg/g dry fraction (Table 3).

**Table 3:** The types of phenolic acids detected in phenolic-enriched fraction (PEF)

Phenolic compounds (mg/g dried PEF)			
Gallic acid	12.7 ± 0.12	Syringic acid	6.9 ± 0.11
Pyrogallol	10.0 ± 0.09	Epicatechin	7.7 ± 0.07
Vanillic acid	7.6 ± 0.14	Catechin	11.5 ± 0.10
Caffeic acid	8.1 ± 0.08	Ferulic acid	13.2 ± 0.08

All data are presented as means (± S.E.M) of at least three replicates (*n* = 3)

*S.E.M* Standard error of the means

### Antioxidant activity

The IC_50_ values presented in Table 4 indicated the antioxidant activity of PEF and gallic acid (positive control). The DPPH scavenging activity, reducing power activity, ß-carotene bleaching inhibition and TBARS inhibition values for PEF were 24.6, 31.2, 37.1 and 42.9 μg/ml and these values were significantly (*p* < 0.05) higher than that of gallic acid with the values of 4.6, 7.4, 11.6 and 14.5 μg/ml, respectively.

**Table 4:** The IC_50_ values indicating antioxidant activity of phenolic-enriched fraction (PEF) and positive control (gallic acid)

	IC_50_ (μg/ml)			
	DPPH scavenging activity	Reducing power activity	ß-carotene bleaching inhibition	TBARS inhibition
PEF	24.6^a^	31.2^a^	37.1^a^	42.9^a^
Gallic acid	4.6^b^	7.4^b^	11.6^b^	14.5^b^
S.E.M	0.82	0.79	1.27	1.12

All data are presented as means (± SEM) of at least three replicates (*n* = 3)

Means (*n* = 3) with different superscripts (a,b) within a column are significantly different (*p* < 0.05)

*S.E.M* Standard error of the means

### Cytotoxic assay

**Fig. 2:**
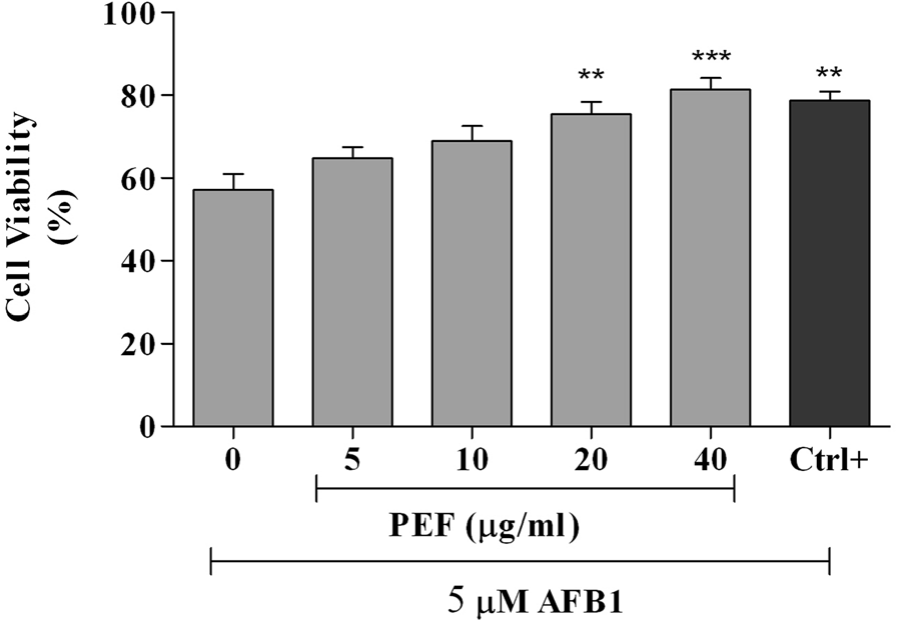
The cytoprotective activity of PEF and gallic acid against AFB1-cell damage. All values are means ± S.E.M of three independent experiments. The cells were pre-treated with different concentrations of PEF and gallic acid as positive control (10 μM or 1.7 μg/ml) for 24 h and then the media were replaced with a medium containing 5 μM of AFB1 and the cells were incubated for another 48 h. The experiment was performed in triplicate. ***p < 0.001 and **p < 0.01 indicated significant difference compared to the untreated control (0)

The cytoprotective activities of PEF and gallic acid against AFB1-cell damage are shown in Fig. 2. The AFB1 at the concentration of 5 μM decreased the cell viability to 57.1 % upon 48 h incubation. Treatment of cells with 20 and 40 μg/ml of PEF enhanced cell viability significantly (*p* < 0.01). Similarly, gallic acid with the concentration of 10 μM or 1.7 μg/ml significantly (*p* < 0.01) improved the cell survival.

### Antioxidant enzymes and lipid peroxidation

Table 5 shows the results of total protein, lipid peroxidation and antioxidant enzymes activity in chicken hepatocytes. It was observed that with the increase in the concentration of PEF, the total cellular protein increased from 0.6 mg/ml to 0.8 mg/ml. The lipid peroxidation values were reduced gradually from 7.5 to 3.8 nM MDA/mg protein. The activities of SOD, CAT and GR were 4.2, 3.8 and 0.2 U/mg protein, respectively, without the addition of PEF, but increased to 7.8, 6.9 and 0.5 U/mg protein, respectively, when cells were treated with 40 μg/ml of PEF. In general, the concentrations of 20 and 40 μg/ml of PEF significantly (*p* < 0.05) improved the oxidative activities of biomarkers, indicating the potential of the PEF to alleviate the negative impacts of AFB1 on hepatocytes functions. Furthermore, these concentrations of PEF exhibited comparable results to that of gallic acid as a reference antioxidant used in this study.

**Table 5:** The total protein, lipid peroxidation and antioxidant enzyme activity of chicken hepatocytes pretreated with phenolic-enriched fraction (PEF) and exposed to AFB1

Items	AFB1 (5 μM)						S.E.M
	PEF (μg/ml)					Control+	
	0	5	10	20	40		
Total protein (mg ml^−1^)	0.6^b^	0.6^b^	0.6^b^	0.8^a^	0.8^a^	0.8^a^	0.02
Lipid peroxidation (nM MDA mg^−1^ protein)	7.5^a^	6.9^ab^	5.6^c^	4.3^d^	3. 8^de^	4.6^d^	0.46
SOD activity (U mg^−1^ protein)	4.2^d^	4.8^cd^	5.7^bc^	6.3^b^	7.8^a^	6.7^ab^	0.8
CAT activity (U mg^−1^ protein)	3.8^de^	4.2^d^	4.9^bcd^	5.7^b^	6.9^a^	5.5^bc^	0.47
GR activity (U mg^−1^ protein)	0.2^c^	0.2^c^	0.4^b^	0.5^a^	0.5^a^	0.5^a^	0.06

All cells were pre-treated with different concentrations of PEF for 24 h, then the media were replaced with a medium containing 5 μM of AFB1 and the cells were incubated for another 48 h

*MDA* Malondialdehyde as lipid peroxidation biomarker

Control+: 10 μM or 1.7 μg/ml gallic acid

*SOD* Superoxide dismutase

*CAT* Catalase

*GR* Glutathione reductase

*S.E.M* Standard error of the means

Means (*n* = 3) with different superscripts (a,b,c,d,e) within a row are significantly different (*p* < 0.05)

### Changes in molecular biomarkers of oxidative stress

The changes in the expression of various genes in chicken hepatocytes are presented in Table 6. The gradual increase in PEF concentration up-regulated the anti-apoptosis gene (bcl2) and down-regulated the expressions of NF-kB, proinflammatory mediators (iNOS, TNF-α, IL1ß and IL6) and pro-apoptotic gene (bax). The PEF at 40 μg/ml produced comparable results to gallic acid as a positive control.

**Table 6:** The changes in the expression of different genes in chicken hepatocytes pretreated with phenolic-enriched fraction (PEF) and exposed to AFB1

	Gene expression (Fold changes)						
Items		PEF concentration (μg/ml)				Control+	S.E.M
	0	5	10	20	40		
Up-regulated genes							
Bcl2	1.0^d^	+2.1^cd^	+3.2^c^	+4.3^b^	+5.9^a^	+5.6^a^	0.39
Down-regulated genes							
NF-kB	1.0^d^	−1.5^d^	−2.6^c^	−3.6^b^	−4.4^a^	−4.6^a^	0.36
TNF-α	1.0^d^	−1.2^cd^	−2.1^c^	−3.7^b^	−5.1^a^	−4.9^a^	0.41
IL1ß	1.0^d^	−1.1^d^	−1.6^c^	−2.2^b^	−2.9^a^	−3.2^a^	0.18
IL6	1.0^d^	−1.3^cd^	−1.7^c^	−2.4^b^	−3.5^a^	−3.4^a^	0.21
iNOS	1.0^d^	−1.7^cd^	−2.4^bc^	−3.3^b^	−4.8^a^	−4.4^a^	0.43
Bax	1.0^d^	−1.8^cd^	−2.7^c^	−4.2^b^	−5.7^a^	−5.2^a^	0.47

The cells were pre-treated for 24 h with different concentrations of PEF ranging from 0 to 40 μg/ml and positive control (10 μM or 1.7 μg/ml gallic acid). The media were replaced with a medium containing 5 μM of AFB1 and the cells were incubated for another 48 h

The expression of each gene was normalised to the GAPDH and ß-actin expressions as housekeeping genes and then the result normalised to the expression of that gene in the negative control (PEF 0 μg/ml)

*S.E.M* Standard error of the means

Means (*n* = 3) with different superscripts (a,b,c,d) within a row are significantly different (*p* < 0.05)

**Fig. 3:**
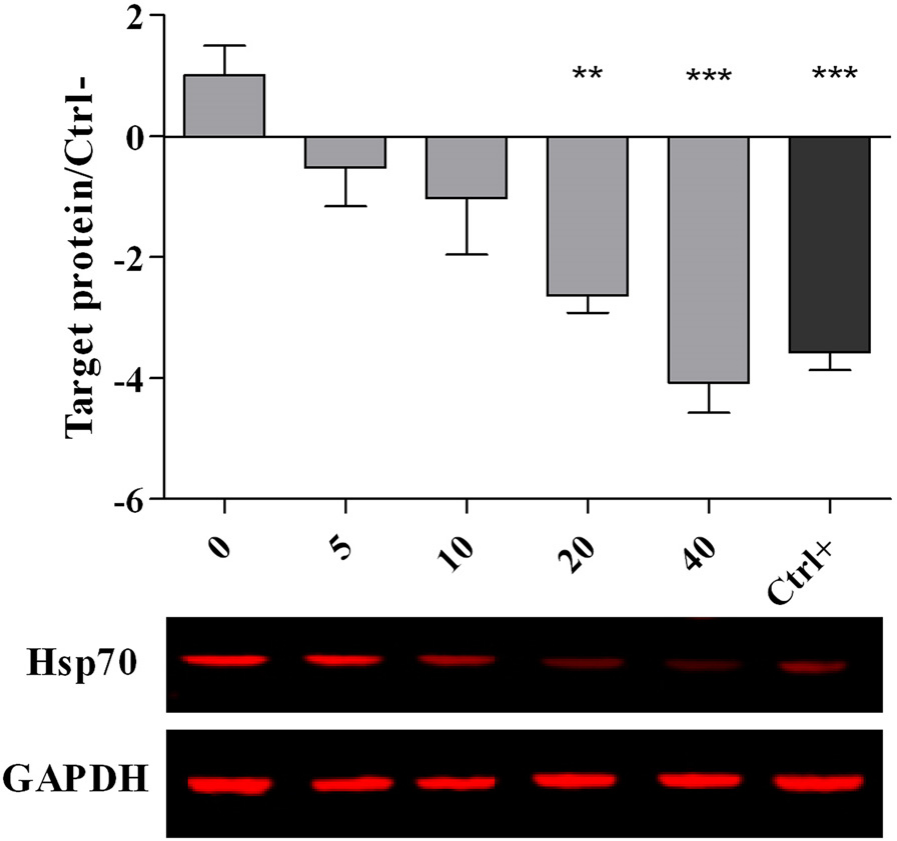
Expression of Hsp70 protein in chicken hepatocytes. The cells were pre-treated for 24 h with different concentrations of PEF ranging from 0 to 40 μg/ml and positive control (gallic acid, 10 μM or 1.7 μg/ml). The media were replaced with a medium containing 5 μM of AFB1 and the cells were incubated for another 48 h. All values represent means ± SEM from three independent experiments. *** *p* < 0.001 and ** *p* < 0.01 indicate significant difference compared to the untreated control (0)

**Fig. 4:**
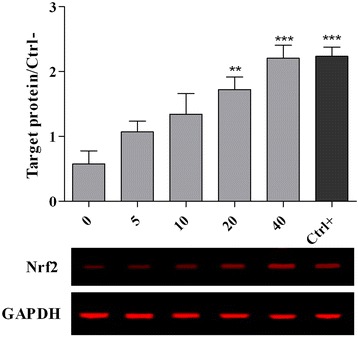
Expression of nrf2 protein in chicken hepatocytes. The cells were pre-treated for 24 h with different concentrations of PEF ranging from 0 to 40 μg/ml and positive control (gallic acid, 10 μM or 1.7 μg/ml). The media were replaced with a medium containing 5 μM of AFB1 and the cells were incubated for another 48 h. All values represent means ± SEM from three independent experiments. *** *p* < 0.001 and ** *p* < 0.01 indicate significant difference compared to the untreated control (0)

**Fig. 5:**
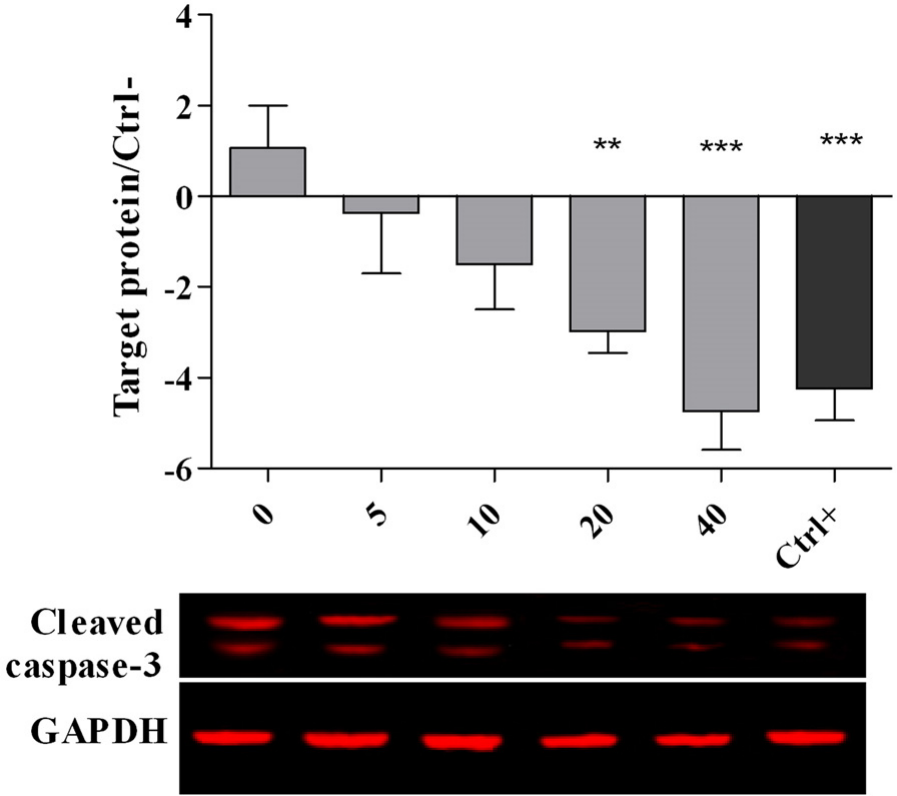
Expression of caspase-3 protein in chicken hepatocytes. The cells were pre-treated for 24 h with different concentrations of PEF ranging from 0 to 40 μg/ml and positive control (gallic acid, 10 μM or 1.7 μg/ml). The media were replaced with a medium containing 5 μM of AFB1 and the cells incubated for another 48 h. All values represent mean ± standard error from three independent experiments. *** *p* < 0.001 and ** *p* < 0.01 indicate significant difference compared to the untreated control

The western blot analysis confirmed the changes in the expression of Hsp70 (Fig. 3), nrf2 (Fig. 4) and caspase-3 (Fig. 5) proteins. It was observed that the high concentrations of PEF (20 and 40 μg/ml) significantly (*p* < 0.05) down-regulated the Hsp70 and caspase-3 proteins while up-regulated the nrf2 protein as compared to the cells without PEF pretreatment (0 μg/ml). The gallic acid has also been found to down-regulate the Hsp70 and caspase-3 proteins and up-regulate the nrf2 protein.

### Apoptosis analysis by flow cytometry

**Fig. 6:**
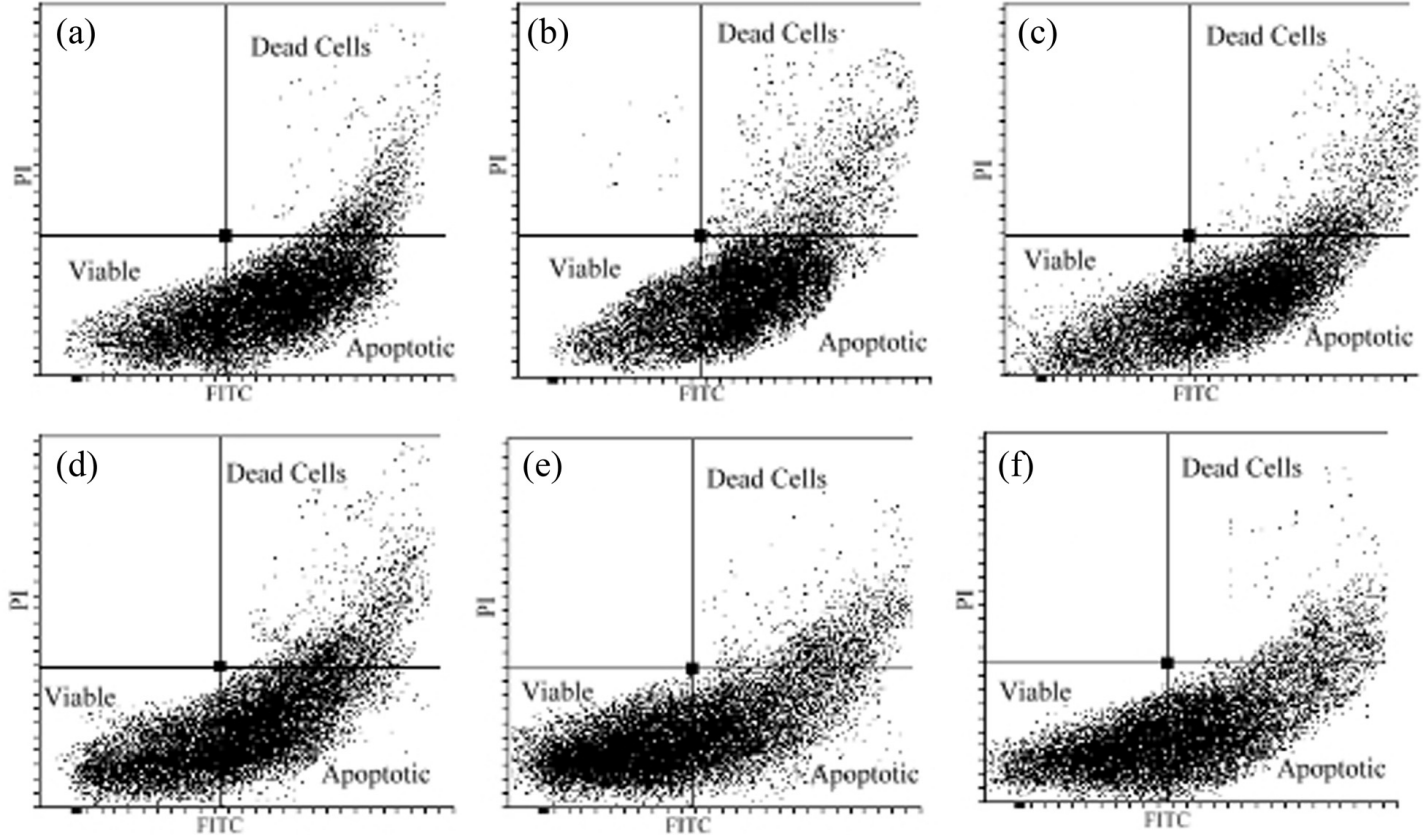
Flow cytometry analyses of chicken hepatocytes pretreated with phenolic-enriched fraction (PEF) and exposed to 5 μM of AFB1. The a, b, c, d and e showed cells pretreated with 0, 5, 10, 20 and 40 μg/ml of PEF, respectively. The f indicated that the cells were pretreated with gallic acid at the concentration of 10 μM or 1.7 μg/ml

The flow cytometry analysis plots are presented in Fig. 6. As observed in Fig. 6-a, the majority of cells showed apoptosis. However, it was evident that the increase in the concentration of PEF resulted in the decrease of the population of apoptotic cells (Fig. 6–b to -e). Similarly, Fig. 6–f indicates that pretreatment of cells with the gallic acid decreased the apoptotic cells. The percentage values for the viable, apoptotic and dead cells obtained from flow cytometry analysis are presented in Table 7. In the medium without PEF (0 μg/ml), the majority of the cell mass (52.8 %) were recognised as apoptotic cells. The increasing concentrations of PEF reduced the percentage of apoptotic cells and enhanced the cell viability significantly (*p* < 0.05). The percentage of apoptotic and viable cells in hepatocytes treated with gallic acid as a positive control were 41.1 and 40.2 %, respectively, which were comparable to that of hepatocytes treated with 20 and 40 μg/ml of PEF.

**Table 7:** Percentage of viable, apoptotic and dead cells analysed by flow cytometry

Cells (%)	PEF (μg/ml)					Control+	S.E.M
	0	5	10	20	40		
Viable	15.2^d^	16.2^d^	27.3^c^	36.1^b^	46.2^a^	40.2^ab^	3.2
Apoptotic	52.8^a^	54.1^a^	48.4^b^	43.4^bc^	37.1^d^	41.1^cd^	2.4
Dead	32.1^a^	29.8^a^	24.3^b^	20.7^bc^	16.9^d^	18.8^cd^	2.1

The cells were pre-treated with different concentrations of PEF for 24 h, then the media were replaced with a medium containing 5 μM of AFB1 and the cells were incubated for another 48 h

A minimum of 15,000 cells per sample was analysed by flow cytometry

Control+: 10 μM or 1.7 μg/ml gallic acid

Means with different superscripts (a,b,c,d) within a row are significantly different (*p* < 0.05)

*PEF* Phenolic-enriched fraction

*S.E.M* Standard error of the means

## Discussion

The types of phenolic compounds detected in PEF were slightly different from that of the oil palm fruit extract which showed the presence of protocatechuic, p-hydroxybenzoic and p-coumaric acids, besides gallic, vanillic, caffeic, syringic and ferulic acids [28]. Tan et al*.* [29] also reported the presence of p-hydroxybenzoic acid, cinnamic acid, ferulic acid and coumaric acid in the oil, while, Jaffri et al*.* [30] observed the presence of catechin derivatives in the oil palm leaf extract. It seemed that the PEF contained additional types of phenolic compounds as compared to those detected in the oil and leaf extracts. However, the types of phenolic compounds present are subjected to numerous factors including extraction, detection and identification procedures, besides other agronomic factors. Phenolic compounds have been reported as a potential antioxidant [31, 32], thereby PEF can be considered a reliable source of natural antioxidants for protecting cells against xenobiotics toxicity.

The results presented in Table 5 and Fig. 2 revealed that, pretreatment of hepatocytes with different concentrations of PEF improved the lipid peroxidation and cellular antioxidant enzymes, concomitant with the increase in cell viability when cells were exposed to AFB1. These findings reflected the cytoprotective properties of PEF, which could be attributed to its antioxidant activity. Previous studies have confirmed the ability of mycotoxins to impair the balance between pro-oxidants and antioxidants in the cells, which resulted in the production of reactive oxygen species (ROS), that led to lipoperoxidation and oxidative stress [3–7, 9, 12]. Consequently, it was emphasized that antioxidant enzymes would function as the main defense mechanism against the ROS in the cells. In the present study, the PEF exerted cytoprotective effect which reduced the toxic symptoms of AFB1 probably through activation of antioxidant enzymes and inhibition of lipid peroxidation chain reaction. Similar studies have reported the role of natural antioxidants in enhancing antioxidant enzymes, inhibiting lipid peroxidation and alleviating oxidative stress in cells exposed to mycotoxins [6, 33–35]. The PEF at 40 μg/ml showed cytoprotection activity and alleviation of oxidative biomarkers reaching up to that of gallic acid at 1.7 μg/ml. Although the concentrations of phenolic compounds used in the assay differ, the PEF should be considered effective in alleviating the AFB1 effects. These findings should be of interest in lieu of PEF source and availability.

**Fig. 7:**
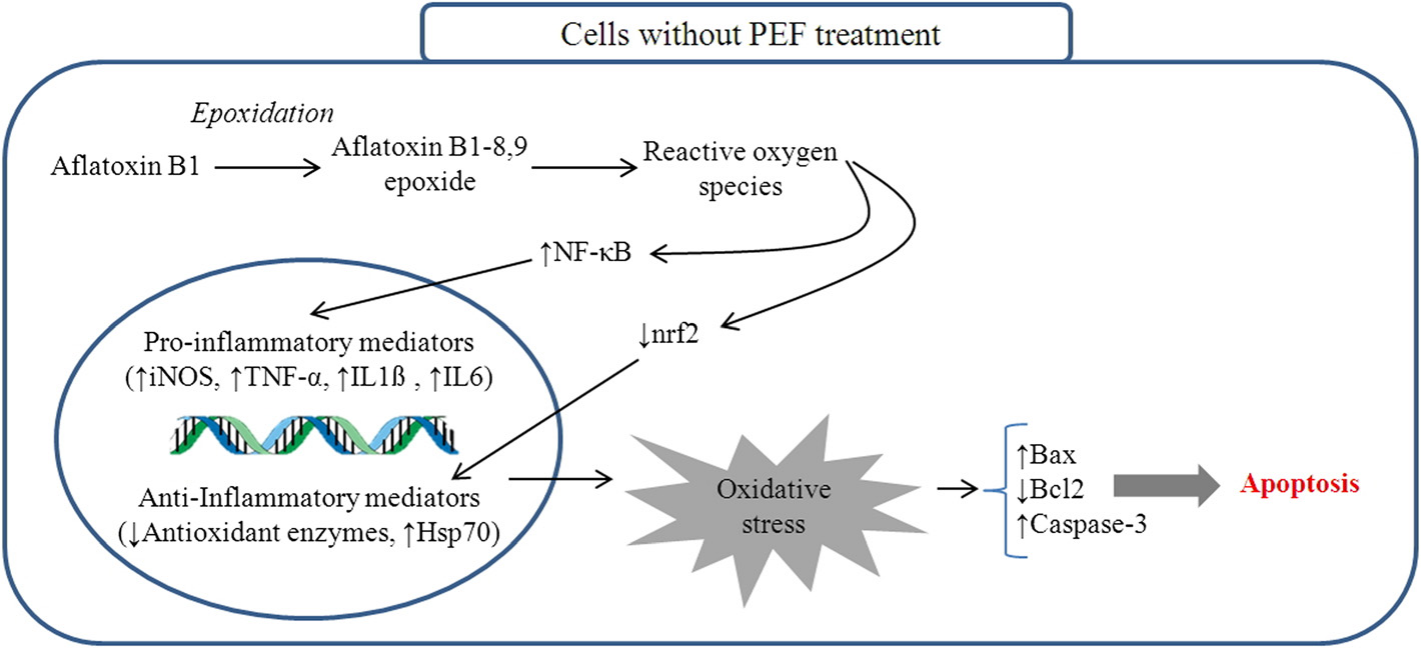
This diagram illustrates the events taking place in un-treated chicken hepatocytes exposed to AFB1

Table 6 and Figs. 3, 4 and 5 show the expression of molecular biomarkers involved in oxidative stress, inflammatory response and apoptosis in cells pretreated with various concentrations of PEF and exposed to AFB1. The relationship between expressions of genes and proteins provides a better understanding of the mechanism of action of PEF against AFB1-cell damage. As shown in Fig. 7, the AFB1 was converted to the Aflatoxin B1–8,9 epoxide through epoxidation and this active form initiated the production of ROS in the cells. Thereafter, the excessive amount of ROS elicited the NF-kB up-regulation and nrf2 down-regulation. The NF-kB upon up-regulation induced the expression of various pro-inflammatory mediators, including iNOS, TNF-α, IL1ß and IL6 (Fig. 7). The nrf2 as the redox-sensitive transcription factor could activate the cellular antioxidant defense mechanism and enhance the production of antioxidant enzymes and heat shock proteins [36]. The down-regulation of nrf2 reduced the antioxidant enzymes production and increased the up-regulation of Hsp70 (Fig. 7). The Hsp70 plays a role as a sensor of cellular redox changes acting like antioxidant enzymes. For instance, under oxidative stress the intracellular components, particularly proteins could undergo oxidation. The Hsp70 restores and maintains the redox homeostasis of the cells even under oxidative stress [36].

**Fig. 8:**
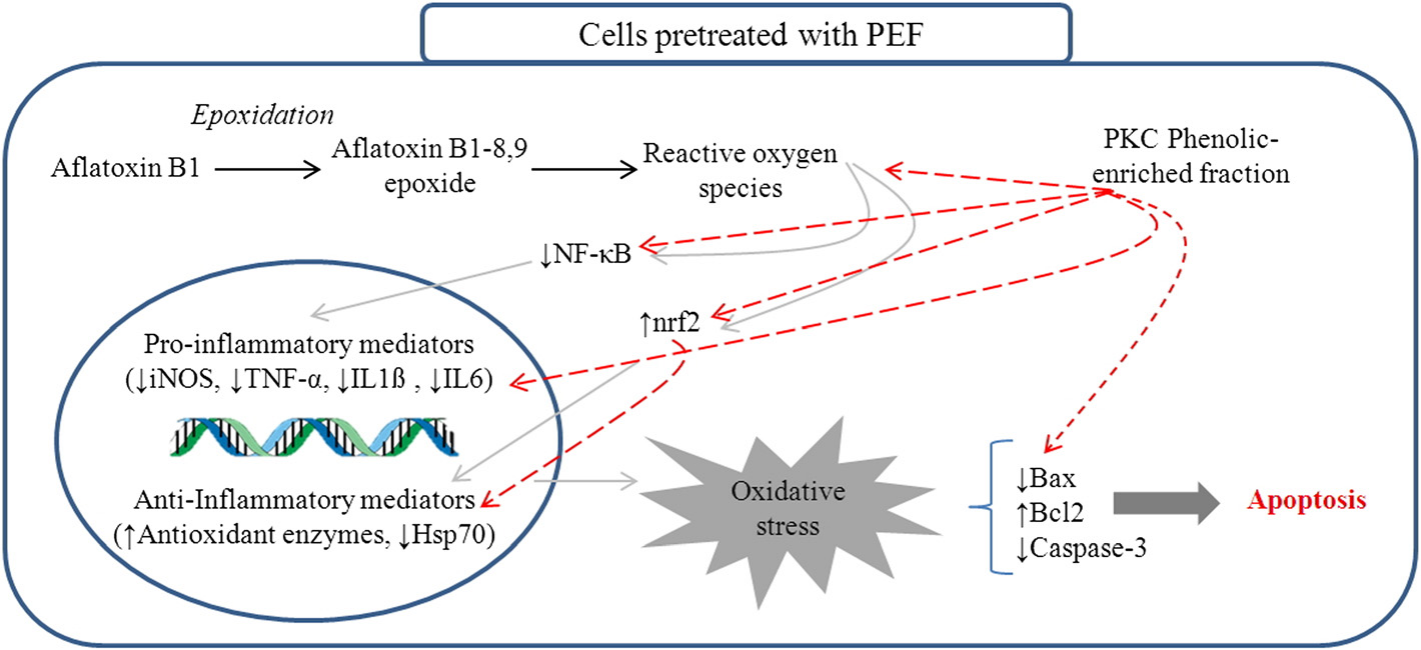
This diagram illustrates the probable protective mechanisms of PEF in chicken hepatocytes exposed to AFB1

The up-regulation of pro-inflammatory mediators accompanied by suppression of anti-inflammatory proteins disrupted the cell homeostasis leading to an imbalance between anti-apoptotic (bcl2) and pro-apoptotic (bax and caspase-3) effectors. The imbalance between anti-apoptotic and pro-apoptotic effectors induced apoptosis and cell death (Fig. 7). The pretreatment of cells with PEF induced the cells to attenuate the expression of NF-kB and subsequently pro-inflammatory mediators (iNOS, TNF-α, IL1ß and IL6) (Fig. 8). This is possibly due to the direct inhibition of ROS together with modulation of NF-kB expression. On the other hand, the suppression of pro-inflammatory mediators through direct action of PEF could be another plausible reason (Fig. 8).

The PEF up-regulated the expression of nrf2 and its activation enhanced the antioxidant enzymes production (Table 5), while inhibiting the Hsp70 expression. The inverse relationship between antioxidant enzymes and Hsp70 protein suggests that the antioxidant enzymes regulate the cellular redox homeostasis in lieu of Hsp70 protein, thereby high expression of Hsp70 is no longer required. In line with this result, Costa et al*.* [37] also reported the critical role of phenolic compounds in up-regulation of nrf2 protein and enhancement of antioxidant enzymes production.

The results of the present study showed that, the PEF not only affected the NF-kB and nrf2 expressions, but also regulated the imbalance between anti-apoptotic and pro-apoptotic effectors resulting in the enhancement of cell survival. The anti-apoptotic activity of PEF was probably due to the presence of phenolic compounds (gallic acid, pyrogallol, vanillic acid, caffeic acid, syringic acid, epicatechin, catechin and ferulic acid) which up-regulated the bcl2 gene and down-regulated the bax gene and caspase-3 protein (Fig. 8, Table 6). Similarly, several observations demonstrated the close relationship between antioxidant activity of phenolic compounds with cellular antioxidant enzymes activity and reduction of apoptosis cell death in various animal and human cell lines [5, 11, 38].

## Conclusions

The present study showed that PEF could be considered as a cytoprotective agent by up-regulating the antioxidant-related genes and down-regulating the pro-inflammatory and apoptosis associated genes in hepatocytes exposed to AFB1. The findings valorised the phenolic compounds of PKC and paved the way for production of an alternative cytoprotective agent against AFB1 cytotoxicity.

## Abbreviations

PKC: Palm kernel cake
AFB1: Aflatoxin B1
PEF: Phenolic-enriched fraction
ROS: Reactive oxygen species
TPC: Total phenolic content
GAE: Gallic acid equivalent
DPPH: 1,1-diphenyl-2-picryl-hydrazil
ACUC: Animal Use and Care Committee
MTT: 3-(4,5-Dimethylthiazol-2-yl)-2,5-Diphenyltetrazolium Bromide
PBS: Phosphate-buffered saline
SOD: Superoxide dismutase
CAT: Catalase
GR: Glutathione reductase
MDA: Malondialdehyde
TBARS: Thiobarbituric acid reactive substance
SDS: Sodium dodecyl sulphate
NF-kB: Nuclear factor kappa-light-chain-enhancer of activated B cells
iNOS: Nitric oxide synthase
TNF-α: Tumor necrosis factor alpha
IL1ß: Interleukin-1 beta
IL6: Interleukin-6
GAPDH: Glyceraldehyde 3-phosphate dehydrogenase
Hsp70: 70 kilodalton heat shock protein
Nrf2: Nuclear factor (erythroid-derived 2)-like 2
